# Manufacturing and clinical applications of non‐CAR‐T immune effector cells

**DOI:** 10.1111/trf.70144

**Published:** 2026-03-06

**Authors:** Thane Kubik, Theodros Mamo, David H. McKenna

**Affiliations:** ^1^ Alberta Precision Labs Calgary Alberta Canada; ^2^ OhioHealth Arthur G.H. Bing, MD, Cancer Center Blood and Marrow Transplant and Cellular Therapy Program Columbus Ohio USA; ^3^ Molecular and Cellular Therapeutics, Department of Laboratory Medicine and Pathology University of Minnesota Medical School Minneapolis/Saint Paul Minnesota USA

**Abbreviations**
4‐1BB: also known as CD137 or TNFRSF (Tumor Necrosis Factor Receptor Superfamily member 9)
aAPC: artificial antigen presenting cell
ADCC: antibody‐dependent cellular cytotoxicity
ADV: adenovirus
APC: antigen presenting cell
APC: antigen presenting cell
ARDS: acute respiratory distress syndrome
BIKE: bispecific killer cell engager
BKV: BK virus
BLA: biologics license application
CAR: chimeric antigen receptor
CD: cluster of differentiation or cluster of designation
cGMP: current good manufacturing practices
CISH: cytokine inducible SH2 containing protein
CMC: chemistry, manufacturing and controls
CMV: cytomegalovirus
CT: cell therapy
CTL: cytotoxic T lymphocyte
CTLA‐4: cytotoxic T‐lymphocyte‐associated protein 4
DC: dendritic cell
DMSO: dimethylsulfoxide
DP: drug product
EBNA1: Epstein–Barr nuclear antigen 1
EBV: Epstein–Barr virus
EGFR: epidermal growth factor receptor
ELIspot: enzyme‐linked immunospot
FACS: fluorescence activated cell sorting
FDA: Food and Drug Administration
FOXP3: forkhead box protein P3
GFR: glomerular filtration rate
GvHD: graft versus host disease
HHV‐6: human herpes virus‐6
HLA: human leukocyte antigen
HSCT: hematopoietic stem cell transplant
ICI: immune checkpoint inhibitor
IEC: immune effector cell
IFN: interferon
IL: interleukin
IND: investigational new drug
iNK: iPSC‐natural killer
IPEX: immune dysregulation, polyendocrinopathy, enteropathy, X‐linked syndrome
iPSC: induced pluripotent stem cell
IRB: institutional review board
JCV: JC virus
KIR: killer cell immunoglobulin‐like receptor
LAK: lymphokine‐activated killer cell
LCL: lymphoblastoid cell line
LMP2: latent membrane protein 2
MSC: mesenchymal stromal cell
NCAM: neural cell adhesion molecule
NK: natural killer
NSCLC: non‐small cell lung cancer
nTreg: natural regulatory T cell
OKT3: Ortho Kung T3
ORR: overall response rate
PBMC: peripheral blood mononuclear cell
PD‐1: programmed cell death protein 1
PLS: passenger lymphocyte syndrome
PML: progressive multifocal leukoencephalopathy
PTLD: post‐transplant lymphoproliferative disorder
pTreg: peripheral regulatory T cell
QC: quality control
REP: rapid expansion protocol
RM: regenerative medicine
RNA: ribonucleic acid
SARS‐CoV‐2: severe acute respiratory syndrome coronavirus 2
SOP: standard operating procedure
TCM: central memory T cell
TCR: T cell receptor
TGF‐beta: transforming growth factor‐beta
Th2: T helper 2 cell
TIL: tumor infiltrating lymphocyte
TM: transfusion medicine
Treg: regulatory T cell
TRIKE: trispecific killer cell engager
tTreg: thymic regulatory T cell
UCB: umbilical cord blood
VST: virus specific T cell

The field of Transfusion Medicine (TM) has evolved over the years to include much more than traditional blood banking. In many institutions TM includes apheresis services and other labs, such as coagulation, HLA, and cell therapy. Cell Therapy (CT), along with Regenerative Medicine (RM), is a complex, highly regulated field, and thus having the lab housed in TM within Laboratory Medicine & Pathology makes sense. While CT is much more complex than traditional blood banking, the “blueprint” and lessons learned from blood banking serve as a framework for operations, allowing labs to meet both standards set by professional organizations (e.g., AABB, FACT) and regulations from FDA and other relevant authorities. Technologists trained in blood banking are often recruited to CT labs, as they are accustomed to using aseptic technique while working with bags/tubing sets to manipulate blood components. Finally, physicians trained in TM and CT lab processing are best prepared to be laboratory and medical directors of CT labs, as their training is directly geared toward directorship of a clinical lab.

In addition to focusing on hematopoietic progenitor cells (HPCs; i.e., marrow, apheresis products, and umbilical cord blood) in support of blood and marrow transplant programs, many CT labs house a translational research and development section. Often the translational lab will play a role in the development of protocol‐specific immune monitoring assays, the preparation of protocol‐specific sample and data collection, review and reporting plans. However, the main responsibility of the lab is the development of novel CT and RM products. Most translational labs will follow this general roadmap: (1) evaluation of research and pre‐clinical studies, (2) scale‐up and optimization, and (3) methods validation.

*Evaluation of research and pre‐clinical studies*. At this stage, the research and pre‐clinical data is evaluated for feasibility to move forward into the clinical arena. This evaluation includes an assessment from several perspectives, including medical, scientific, technical and regulatory. The results of toxicology and ‘proof of concept’ studies, including studies of cell survival kinetics and distribution, are reviewed. The overall goal is to define the desired characteristics of the cellular product, establish possible methods of cell manufacturing and product testing, and identify appropriate instrumentation and optimal reagents. Often, the reagents and instrumentation used in early studies are typically not suitable for human use and are of smaller scale, respectively. Project management commences with timelines and milestones established, and a pre‐IND meeting/call may occur at this stage as well.

*Scale‐up and optimization*. Prior to the initiation of the clinical trial, development of a reproducible, large‐scale and clinically relevant methodology is required. A technology transfer from the research‐based lab to the translational development lab occurs. Frequently, research‐based methods are not refined, lacking standard operating procedures (SOPs) and details that would allow smooth transition into current good manufacturing practices (cGMP) manufacturing. One obvious example required for this stage is the determination of culture requirements (e.g., media type, components, vessel selection, media changes and other requirements). Confirmation of seeding density and media depth requirements will guide culture vessel selection. Once the details for culture are determined, SOPs and a batch production record (BPR) can be developed.

*Methods validation*. The validation phase begins when an optimized and clinically appropriate method has been defined. The SOPs and BPR are established, and a validation plan is written and then executed. Typically, a validation consists of three successive runs which must pass pre‐established specifications; often most or all of these specifications become the product lot release criteria. The Chemistry, Manufacturing and Controls (CMC) section [i.e., the product manufacturing methods which accompanies the investigational new drug (IND) application] can now be finalized by the translational lab. The CMC and validation summary are provided to FDA in support of the IND application and, in some cases, the Institutional Review Board (IRB) submission. Following acceptance by the FDA, or other relevant authority if outside the USA, this process leads to the cGMP production of the novel therapy in support of a phase I clinical trial. Figure 1 provides an overview of the general manufacturing schema for cellular therapies.

**FIGURE 1:**
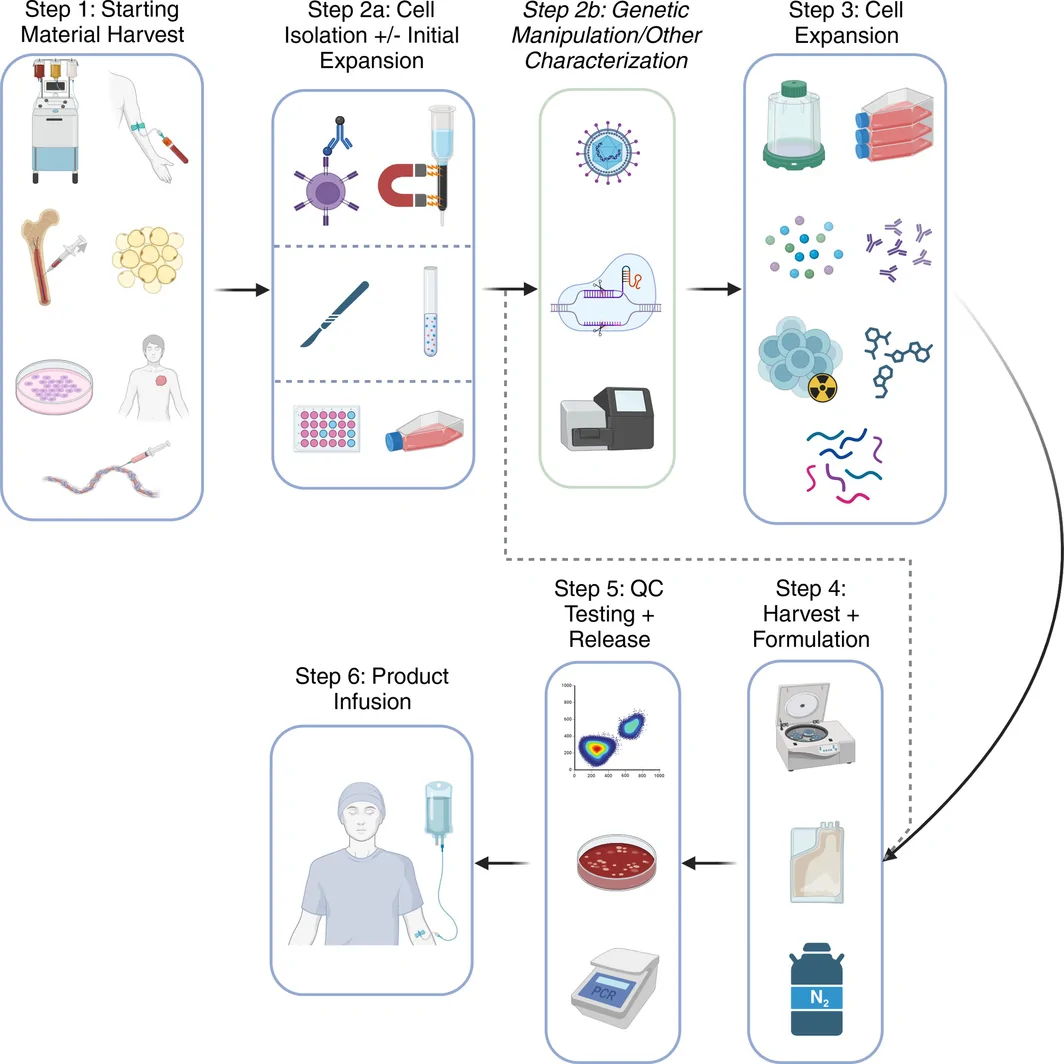
General cell manufacturing schema for immune effector cell therapy. *Step 1*. The manufacturing of these cell therapy products begins with the procurement of starting material from cells and tissues including (clockwise from top left): PBMCs from leukapheresis, whole blood, adipose tissue, tumor tissue, UCB, cell culture/master cell banks (e.g., iPSC) and bone marrow. *Step 2a*. In the cell processing lab, cells of interest undergo selection with or without preliminary expansion. Common selection techniques include positive/negative immunomagnetic column isolation (top), as well as physical and enzymatic isolation from surrounding tissues (middle). Some protocols involve initial expansion followed by cell selection based on target characteristics (e.g., INFγ release), while others rely on specific media and culture characteristics (e.g., cytokines). Some protocols (e.g., direct selection of VSTs) may skip forward to cell harvest and DP formulation (step 4) at this point. *Step 2b*. This optional step involves genetic manipulation using tools such as lentiviral vector transduction (top) or CRISPR/Cas9 gene editing (middle). More information may also be gained to inform DP targets. For example, tumor sequencing (bottom) of neoantigens guides target peptide synthesis in the next step. *Step 3*. There is wide variation in culture materials and reagents to expand cells of interest (clockwise from top left): Different bioreactors and culture flasks can be employed along with the addition of agonistic antibodies (e.g., anti‐CD3/CD28), small molecules (e.g., nicotinamide for NK cells, rapamycin for Tregs), peptides (e.g., viral pepmixes for VSTs, tumor neoantigens for TILs), irradiated aAPCs, and various cytokines (e.g., IL‐2, IL‐4). *Step 4*. Cells are harvested and may be washed (top), prior to final formulation and fill of the DP (middle), which may be optionally cryopreserved (bottom). *Step 5*. QC testing and lot release involves a series of assays assessing identity (e.g., flow cytometry; top), viability, sterility (e.g., microbiological cultures and mycoplasma PCR; middle and bottom), purity (e.g., endotoxin) and potency (e.g., INFγ release). *Step 6*. A fresh or thawed cryopreserved product is administered to a patient who may or may not receive preparatory (e.g., lymphodepletion chemotherapy) and/or post‐infusion (e.g., IL‐2 infusions) drug regimen(s). aAPCs, artificial antigen presenting cells; DP, drug product; iPSC, inducible pluripotent stem cells; NK, natural killer cells; PBMCs, peripheral blood mononuclear cells; PCR, polymerase chain reaction; QC, quality control; Tregs, regulatory T‐cells; TILs, tumor‐infiltrating lymphocytes; UCB, umbilical cord blood. “Created in BioRender. Kubik, T. (2025) https://BioRender.com/ti1cl0q.”

Currently in the USA there are 46 licensed cell and gene therapies (https://www.fda.gov/vaccines‐blood‐biologics/cellular‐gene‐therapy‐products/approved‐cellular‐and‐gene‐therapy‐products; accessed June 10, 2025).^1^ A growing number of these products are immune effector cells (IECs), now primarily chimeric antigen receptor (CAR)‐T cells. While dendritic cells [Provenge (Dendreon) approved in 2010 for prostate cancer] and T cell receptor (TCR) T cell therapies [Tecelra (Adaptimmune) approved in 2024 for synovial sarcoma] are also licensed, here we will focus on a few of the other types of IECs, some of which are represented on the list of licensed products (e.g., Amtagvi, Iovance Biotherapeutics, Inc.), and others that are at various stages of development on the pipeline toward biologics license application (BLA).

## 1 NATURAL KILLER CELLS

### 1.1 Biology

Natural killer (NK) cells were independently described by Rolf Kiessling and Ronald B. Herberman in the 1970s and subsequently named for their ability to kill malignant and infected cells without prior sensitization.^2^, ^3^, ^4^ This marrow‐derived special subset of lymphocytes plays a critical role in the innate immune system. Since the discovery and early understanding of NK cells, their biology and function have been shown to be quite complex with their functional fate (i.e., cytotoxicity) being determined by cumulative inputs (i.e., inhibitory and activating) from interaction with their potential target and other cells, as well as the environment. NK cells lack markers for B and T cells, and they are often characterized at a basic level as being CD3 (pan T cell marker)‐negative and CD56 (isoform of neural cell adhesion molecule, NCAM)‐positive. They account for 5%–15% of circulating lymphocytes and have the morphology of the “large granular lymphocyte”. NK cells are non‐phagocytic and are cytotoxic to IgG‐coated targets, specializing in antibody‐dependent cellular cytotoxicity, or ADCC. They have two approaches to cell killing within the ADCC paradigm—the granule exocytosis pathway (i.e., perforin/granzyme) and the death receptor pathway (i.e., Fas/Fas ligand).^5^ NK cells are a logical selection for both anti‐cancer^6^ and anti‐viral purposes.^7^

### 1.2 Starting material

Current approaches to NK cell manufacturing utilize a variety of sources as starting material. The apheresis peripheral blood mononuclear cell (PBMC) product is likely most common.^8^ However, umbilical cord blood (UCB)^9^ has been used, and a growing number of alternatives (e.g., induced pluripotent stem cells (iPSCs) and NK 92, a lymphoma cell line) offer some exciting promise.^10^, ^11^ Although autologous starting material has been used, most starting material is obtained from allogeneic sources, often haploidentical.^6^ As interest in establishing off‐the‐shelf therapies increases, more sources and different approaches to manufacturing are being considered.

### 1.3 Manufacturing

The earliest experience with NK cells in clinical trials actually involved so‐called lymphokine activated killer (LAK) cells.^12^ Most such approaches involved collection of PBMCs, no further manipulation, and a simple incubation with IL‐2. With the availability of immunomagnetic selection devices, the next generation of manufacturing involved depletions and/or enrichments to better purify the NK cells before incubation and activation with IL‐2 or Il‐15.^8^ Some groups have focused on depletions (e.g., CD3 and CD19 depletions) to avoid potential negative consequences of accessory cells (i.e., T cell‐mediated graft‐vs‐host disease (GvHD) and B cell‐mediated passenger lymphocyte syndrome (PLS) or EBV reactivation/post‐transplant lymphoproliferative disease (PTLD)).^6^, ^13^ Others have found success employing enrichment strategies by including CD56 selection to highly purify the starting material and then move forward into culture.^14^ Some protocols include other cytokines or combinations of cytokines (e.g., IL‐12, IL‐18, and IL‐21). Cytokine induced memory‐like natural killer cells are a type of NK cells with the capacity to produce IFNγ on restimulation, passing this ability down to generations of offspring. These cells require IL‐12, IL‐15, and IL‐18 in the expansion culture.^15^

As it became apparent that cell dose mattered for clinical efficacy, investigators considered ways to increase cell production through culture expansion protocols. PBMC‐derived NK cells can be moderately expanded using nicotinamide^16^ and massively expanded using membrane bound IL‐21 expressing artificial antigen presenting cells (aAPCs).^17^, ^18^ Researchers continue to identify ways to massively expand NK cells while limiting senescence and maintaining or even increasing cytotoxic function. With this in mind, more recently, NK cells have been manufactured using iPSCs. Induced pluripotent stem cells can be made by reprogramming a variety of different cells, including foreskin fibroblasts.^19^ One group has used a two‐stage culture system to efficiently produce NK cells from iPSCs. The first stage involves differentiating iPSCs to CD34+ hematopoietic progenitor cells (HPCs), and the second stage is focused on NK cell differentiation from these iPSC‐derived CD34+ cells (iCD34). The process takes roughly 10 days to move from iPSCs to iCD34 and then about 34 days to differentiate to iPSC‐derived NK cells (iNK).^20^

### 1.4 Testing

In addition to donor eligibility testing, quality control (QC) testing for NK cell products includes the typical testing for novel CT products, such as viability, sterility, Gram stain, and endotoxin. Mycoplasma testing is also included if the manufacturing goes beyond an overnight incubation/activation. Flow cytometry plays a critical role in identifying and quantifying the final “drug product” (DP), often as CD3‐negative/CD56‐positive. It is also critical to characterize any non‐NK cells in the product. At a minimum, this would include T cells (CD3‐positive, possibly looking at CD4 and CD8) and B cells (CD19‐positive) for safety—potential for GvHD and PLS or EBV‐related complications, respectively.^6^, ^13^ For NK cells, the functional assay, which may also be the potency assay, interrogates the capacity for cytotoxicity. Earlier trials included chromium release assays, but other assays, including flow cytometry‐based approaches are now used as well. There is often in process testing and a stability program will include QC testing, typically the testing mentioned above. Many of the QC tests noted are ultimately also lot release testing. NK cell products involving genetic modification (e.g., CAR‐NK) or derivation from iPSCs will undoubtedly have many more QC tests included in the manufacturing process as well as for lot release.^21^

### 1.5 Clinical applications

Despite early evidence indicating autologous primary unmodified NK cells would not be the most efficacious option,^22^ the first approaches to NK cell trials did utilize autologous PBMC from blood or apheresis collections as the starting material.^23^ Discoveries including the identification of killer cell immunoglobulin‐like receptors (KIR) and haploidentical HPC donors and outcomes with transplant furthered our understanding of NK cell function.^24^ Thus, later trials, focused on related haploidentical apheresis PBMC as the starting material, showed promise with substantial clinical results.^6^ Current and future approaches to harnessing NK cell function in the treatment of human disease include: use of NK cells with monoclonal antibodies (ADCC), bispecific or trispecific killer cell engagers (BIKEs, TRIKEs), CAR‐NK or other genetically modified NK cells utilizing starting material such as UCB, NK92, and iPSCs.^11^, ^25^, ^26^ While CAR‐T cells have shown exceptional success in treatment of several diseases, NK cells offer some potential advantages. As compared to an allogeneic CAR‐T, allogeneic NK cells would not be a concern for GvHD. While the benefits of long‐term persistence of CAR‐T are clear, there are rare negative consequences.^27^ NK cells have a short lifespan, and this could be advantageous, yielding clinical effect without long‐term harmful effects. Tumor escape is also potentially less likely with an NK cell treatment. Finally, if an NK cell product can be made as a third‐party, off‐the‐shelf therapy, there would be less logistics issues, cells would be readily available for timely administration, and they would be more cost‐effective.

## 2 REGULATORY T CELLS

### 2.1 Biology

Regulatory T cells (Tregs) are a subset of T cells that comprise 5%–10% of the CD4^+^ T‐cell population and specialize in suppressing the activation and expansion of lymphocytes.^28^ Their key feature is expression of the transcription factor forkhead box protein P3 (FOXP3),^29^, ^30^ along with high surface expression of CD25 and low expression of CD127,^31^ with CD4^+^CD25^+^CD127^low^ T cells marking the key phenotypic characteristic of Tregs. An important milestone in Treg therapy was the discovery of the gene that is essential for the development of functional Tregs, which was first discovered in mouse as the *Foxp3* gene.^32^ Mutations in FOXP3 in humans was found to lead to a severe autoimmune polyendocrine syndrome,^33^ immunodysregulation polyendocrinopathy enteropathy X‐linked syndrome (IPEX)—a syndrome that leads to features of autoimmunity such as enteropathy, endocrinopathy, and dermatitis, as well as autoimmune hepatitis.^34^

Tregs originate in vivo either through direct differentiation in the thymus or develop from conventional CD4^+^ T cells in the periphery. Tregs that originate in the thymus [thymic Tregs (tTregs), previously called natural Tregs (nTregs)], are immature CD4 cells that receive T‐cell antigen receptor (TCR) signals and have high affinity for self‐peptides.^35^ Peripheral Tregs (pTregs) originate in the periphery when conventional CD4^+^ T cells encounter antigens in the presence of specific factors such as TGF‐β.^35^, ^36^ tTregs and pTregs are similar in function with no protein marker to differentiate them in humans.^36^ Tregs function by targeting T cells either directly or indirectly by modulating APC.^37^ Direct cell contact mechanisms involve the expression of negative regulatory cell surface receptors such as cytotoxic T lymphocyte antigen 4 (CTLA‐4),^37^ whereas indirect mechanisms involve the release of anti‐inflammatory soluble mediators such as IL‐10, TGF‐β, and IL‐35.^35^, ^36^, ^37^ Understanding these mechanisms is critical in the design and manufacturing of Treg therapy.

### 2.2 Starting material

The two major sources of Treg products generated under cGMP for clinical trials are PBMCs collected by apheresis and UCB. As Tregs comprise only 5%–10% of the CD4^+^ T cell population, obtaining a pure population of Tregs from PBMCs requires multiple steps of isolation and expansion. In addition to being a direct source of Tregs, PBMCs also provide conventional Tcells that could be driven to a Treg phenotype and function in the context of the right cocktails including TGF‐β, IL‐2 and rapamycin, leading to the development of induced Tregs (iTregs).

While relatively fewer clinical trials have used UCB as the source of Tregs, the potential of third‐party UCB units to be used as an allogeneic source makes them very attractive. UCB also requires advanced techniques for isolating and expanding Tregs to achieve a sufficient dose. However, isolation of UCB‐derived Tregs using CD25 is much more straightforward when compared to PBMC, as the vast majority of CD25^+^ cells in this naïve starting material are Tregs. CD25 is an activation marker, and in PBMC this means most CD25^+^ cells are activated conventional T cells. While Tregs derived from PBMCs and UCB account for most of the clinical trials so far, alternative sources of Tregs for allogeneic products have also been explored. These include pediatric thymuses, removed during pediatric cardiac surgeries, and differentiation of iPSC into Tregs.^38^

### 2.3 Manufacturing

After acquiring the starting material, the key steps of Treg manufacturing involve cell selection/isolation, stimulation/activation, expansion and purification, followed by testing/analysis to make sure the product meets release criteria. For next generation Treg therapy, gene modification/editing is also becoming an important step of the manufacturing process. The most common method of isolating Tregs is using immunomagnetic selection with CD25 as the main surface marker for positive selection. cGMP‐grade fluorescent‐activated cell sorting (FACS) has also been used to isolate Tregs, with cell surface markers such as CD4 and CD25 used for positive selection and CD127 for negative selection.^39^ cGMP‐grade FACS sorting is still in its early stages compared to established immunomagnetic selection.

The two most common approaches for stimulating/activating these cells have been antibodies bound to beads or cell‐based aAPCs. Anti‐CD3‐/anti‐CD28‐coated microbeads are widely used despite the requirement of removing these beads prior to infusion.^40^ The most widely used aAPCs to activate Tregs are K562 cells expressing CD86 and CD64 (KT86/64) that are loaded with anti‐CD3 mAb.^41^ These cells are irradiated prior to use and disappear from culture over time, hence do not require a removal step prior to infusion. The cytokine IL‐2 and immunosuppressive drug rapamycin are the most used agents to expand Tregs.^40^ For Tregs derived from UCB, multiples round of expansion are necessary to achieve high dose.^41^

The ex vivo expansion of Tregs is an important element of the manufacturing step that determines the scalability of the process. The reagents and consumables utilized during this step such as specialized media, cytokines and drugs need to be scaled up and need to be cGMP compliant. The expansion protocols will also need to be scaled up, and validation of the key steps need to take place. The addition of gene modification/editing in the next generation of Tregs makes this even more complex and needs to be considered in the plan to scale the manufacturing steps.

The final step of the Treg manufacturing process is the purification and analysis of the manufactured product, which involves removal of unwanted components such as the various beads used in isolation/activation of the Tregs. The purification of the final product is essential in ensuring the quality of the Tregs to be infused into the patients and is another important step to consider in scaling the manufacturing process. The final product will then need to be tested following the procedures discussed below before final release for patient administration.

### 2.4 Testing

Product release testing and quality control of Tregs, as with all CT products, includes assays for identity, sterility, viability and purity of the product. Given the variability in starting material and manufacturing process, acceptance criteria for the purity level are variable. Purity level is determined mainly through testing of FOXP3 expression and co‐expression of markers such as CD4 and CD25. Sterility and identity are performed using the standard analytical methods. Testing the potency of Treg products is probably the most difficult of all the product release testing. As the understanding of Treg function evolves, the key factors that correlate with immune suppression continue to be elucidated. In vitro suppression assays that measure inhibition of effector T cells and measurement of levels of suppressive cytokines (such as IL‐10, IL‐35 and TGF‐β) as well as inhibitory molecules (such as CTLA‐4) are examples of potency assays explored so far.^38^

### 2.5 Clinical applications

The most common indications that have been explored in the use of Tregs include GvHD, organ transplantation and autoimmune disease such as multiple sclerosis and type I diabetes. Most of the studies have been phase I or II studies investigating the safety and biological activity of the Tregs, with limited phase IIb or III studies so far. While multiple phase I studies exist for organ transplantation and autoimmune disease,^39^ the most advanced studies in the use of Tregs has been for treating GvHD, with recent promising phase III studies.

The use of fresh purified allogeneic Tregs along with conventional T cells to prevent GvHD during an allogeneic hematopoietic stem cell transplant for various hematologic diseases has shown promise in phase Ib studies and a recently completed phase III study.^42^, ^43^ The phase II efficacy study investigating the use of the donor Tregs to prevent GvHD showed a significantly reduced incidence and burden of GvHD and improved GvHD‐free relapse‐free survival compared with the use of unmanipulated donor grafts and multiagent immune suppression.^44^ According to the sponsoring company Orca Bio, the stem cell plus Treg combination product (termed Orca‐T) resulted in positive phase III data and the company has submitted a biological licensing application with expected decision from FDA in April 2026,^45^ possibly opening the door for the first FDA approved Treg product.

Additionally, a phase II/III study of an autologous Treg/Th2 hybrid T cell product in the treatment of amyotrophic lateral sclerosis (ALS) is currently underway through a sponsorship by another pharmaceutical company (NCT04220190). These late‐stage clinical trials in Treg application, along with several promising previous phase I studies, show the excellent potential of Tregs for various clinical applications that could materialize within the next few years.

## 3 MESENCHYMAL STEM OR STROMAL CELLS

### 3.1 Biology

Mesenchymal stem or stromal cells (MSCs) are diverse population of cells with low immunogenicity that have a common property of the ability to secrete paracrine factors such as growth factors, cytokines, chemokines, exosomes and antimicrobial peptides.^46^ The ability to secret these various biological factors gives MSCs the capacity to modulate the immune response, reduce inflammation and support tissue repair by promoting cell‐to‐cell interactions and cellular proliferation.^47^ These properties have made MSCs attractive for applications in treating various diseases such as GvHD, cardiovascular disease and acute respiratory distress syndrome (ARDS).^48^, ^49^, ^50^

Given the various sources of MSCs, their diverse properties, and a lack of consensus among researchers, it was difficult to arrive at universally accepted common characteristics to define MSCs. The Mesenchymal and Tissue Stem Cell Committee of the International Society for Cell and Gene Therapy (ISCT) proposed a set of minimal criteria to define human MSCs. According to this position statement, three criteria are used to define MSCs: adherence to plastic in standard culture conditions, specific surface antigen expression and multipotent differentiation potential.^51^ Antigen expression should be positive for CD105, CD73 and CD90 and negative for CD45, CD34, CD14 or CD11b, CD79a or CD19 and HLA class II, with differentiation potential to osteoblasts, adipocytes and chondroblasts. Since publication of the position statement in 2006, the majority of MSC‐based cell therapies in clinical studies have adhered to the criteria, though additional testing, especially appropriate functional/potency testing, has often been included.

### 3.2 Starting material

MSCs that display the common properties discussed above are derived from various tissue sources. However, the most common sources of MSCs include bone marrow, adipose tissue and UCB.^52^ Bone marrow was the first source of MSCs that led to their discovery, and it is still widely used as a source for clinical applications. Additionally, the effort to enhance the proliferative capacity of MSCs has led to the derivation of MSCs from induced pluripotent stem cells (iPSCs).^53^ Obtaining starting material from all these various sources must be done following aseptic techniques to allow for safe, pure products that meet the criteria specified above.

### 3.3 Manufacturing

Regardless of the source type, the key manufacturing steps for MSCs include culturing the cells to produce a source or master cell bank and then possibly working cell banks, then culturing and expanding the cells at large scale, harvesting, and concluding with final formulation and typically cryopreservation to await infusion.^54^ There is significant variability in these processes as there is no standardized protocol for culturing and harvesting MSCs. The culture media formulations, medium supplementation, initial seeding densities, the number of passages, and the length of time MSCs are maintained in culture and frozen are all variable across the MSCs investigated in various clinical trials.^55^ Even when the source material is the same, these manufacturing variabilities lead to differing viabilities and global gene expression profiles of the MSCs.^56^

For large scale clinical applications, particularly for allogeneic MSC products, an important aspect of the manufacturing process is the ability to scale up the culturing, expansion and harvesting of the cells in high doses capable of being infused into multiple patients. Therefore, the selection of appropriate culture systems and bioreactors is crucial for successful clinical translation.^54^ Additionally, successful cryopreservation of the cells for storage prior to patient administration is a critical step. While DMSO continues to be the standard cryoprotectant for storage of various cellular therapies, novel agents such as those made with sugar, sugar alcohols, and amino acids are also being explored as alternative solutions to freeze MSCs.^57^

Despite the large number of clinical trials over the years, translating MSCs has been slow due to several challenges, including some aspects of the manufacturing steps. The finite expansion capacities, limited lifespan and progressive alterations in their biological properties during in vitro culture are among the challenges experienced.^58^ Considering the inherent heterogeneity of MSCs along with the donor‐to‐donor variability and limitations in expansion potential is critical while designing manufacturing protocols that will lead to effective clinical translation. While identifying effective potency assays has been a bottleneck in the MSC field, improving and scaling up the various manufacturing steps will also be critical in advancing MSC clinical translation.

### 3.4 Testing

QC and release testing for MSCs includes viability, sterility and purity of the product. Additionally, immunophenotyping of the products to ensure the cells meet the minimum criteria for MSCs is important. One of the most difficult aspects of MSC testing that has hampered clinical translation are potency assays. Given the variabilities in source material and manufacturing process, as well as the multiple mechanisms through which MSCs work, potency testing has been a bottleneck for the field of MSC therapy. Ensuring an effective potency assay is developed for each unique application in consultation with the FDA early in the process will be critical in continuing to advance the field.

### 3.5 Clinical applications

While over 1700 MSC‐related clinical trials have been completed over the years,^58^ the first FDA approval of an allogeneic MSC product, which was for the treatment of steroid refractory acute GvHD in pediatric patients, took place in December 2024.^59^ Following the failure of previous phase III trials in the USA, this was a significant achievement in the field. The large multicenter phase III studies for the approved product were conducted over many years and required several resubmissions of biological licensing applications before the final approval.^60^, ^61^ Despite previous approvals in other parts of the world, the FDA approval in the USA is expected to propel the MSC field forward.

Since the first report of successful treatment of a case of severe acute GvHD with an allogeneic MSC product in 2004,^62^ two decades elapsed before the first approval of an MSC product in the USA, mainly due to issues with the potency assay and difficulty showing the efficacy of MSCs in controlled trials. Investigations of patient stratification approaches, critical quality attributes (CQAs) and selection of therapeutically potent MSCs have been identified as key aspects in ensuring successful clinical trial design for future studies.^63^ Patient clinical phenotype and molecular endotype heterogeneity, heterogeneity in the treatment centers administering MSCs, donor‐to‐donor and batch‐to‐batch heterogeneity in manufacturing MSCs, correlative and clinically predictive potency attributes, frequency of dosing, and duration of effects remain significant challenges that need to be overcome to advance the field.^59^ Emboldened by the first FDA approval, there is expectation that these significant challenges will continue to be overcome ensuring that the early promise of MSCs will come to fruition.

## 4 VIRUS SPECIFIC T‐CELLS

### 4.1 Biology

Allogeneic hematopoietic stem cell transplantation (allo‐HSCT) and solid organ transplantation (SOT) require long‐term immunosuppression that predisposes patients to chronic and refractory viral infections. These infections cause significant morbidity and mortality in the post‐transplant period. Viral reactivation typically occurs within the first 6 months post‐transplant, and the most common viruses include cytomegalovirus (CMV), Epstein–Barr Virus (EBV), BK poliomavirus (BKV), adenovirus (ADV) and human herpesvirus 6 (HHV‐6).^64^

The treatment of post‐transplant viral infections remains challenging due to limited availability of effective, targeted antiviral agents with acceptable safety profiles.^65^, ^66^ Antigen‐specific virus specific T‐cell (VST) immunotherapy aims to provide a targeted therapeutic alternative. The first use of a VST occurred in 1992 with the adoptive transfer of donor‐derived CD8+ T‐cells expanded in the presence of CMV‐infected fibroblasts to treat three allo‐HSCT patients.^67^ The adoptive transfer of VSTs has since been expanded to treat hundreds of HSCT and SOT patients with opportunistic viral infections and EBV‐associated post‐transplant lymphoproliferative disorders (PTLD).^65^, ^68^, ^69^, ^70^

Work characterizing effective subsets of T‐cells within VST products has shown a role for both CD8+ cytotoxic T lymphocytes (CTLs) and CD4+ helper cells in viral clearance through antigen‐specific killing, cytokine secretion, and support for endogenous immunity.^71^, ^72^ Products containing exclusively CD8+ cells may show a limited duration of immune response compared to those that include CD4+ cells as well.^73^ VST products with naive T‐cell populations derived from UCB have also been shown to be safe and effective.^68^ Products comprised of T cells with a central memory (TCM) phenotype demonstrate superior in vivo persistence.^74^, ^75^ Challenges to successful treatment with VSTs include viral immune evasion via mutation or HLA downregulation, and T‐cell exhaustion.^76^, ^77^

### 4.2 Starting material

Both autologous and allogeneic sources of starting material for VST manufacturing have been used. Allogeneic donors are typically seropositive for the virus(es) in question. Seropositivity for CMV and EBV should be confirmed in allogeneic donors; however, as there is near universal seroprevalence of ADV, BKV and HHV‐6 in adults, seropositivity is often assumed.^65^ In the context of allo‐HSCT, VSTs have been produced from the original HSCT donor,^66^ but can also be collected from a separate seropositive donor. Alternatively, “off‐the‐shelf” cryobanks of VSTs derived from healthy allogeneic donors offer rapid access to treatment.^65^, ^70^

Peripheral blood mononuclear cells (PBMC) for VST manufacture are typically collected by non‐mobilized leukapheresis. Whole blood draws followed by PBMC isolation via Ficoll gradient separation have also been used.^66^, ^78^ Finally, VSTs can be manufactured from UCB.^68^, ^79^

Autologous sources of VSTs have the advantage of being HLA‐matched and having no risk of GvHD, but they may be suboptimal for manufacture depending on how heavily a patient has been treated with chemotherapy and/or immunosuppressants. VST products derived from allogeneic sources are frequently mismatched at one or more HLA loci. VSTs show an excellent toxicity profile with rare occurrence of GvHD, even with multiple HLA mismatches.^80^, ^81^, ^82^ At the same time, some HLA matching is desirable as there is evidence of preferential class I presentation of EBV/CMV peptides and class II presentation of ADV/BKV peptides.^71^, ^72^, ^82^

In vivo persistence may also be related to the degree of HLA matching, where third party VSTs with high levels of HLA‐mismatch are often cleared within a few weeks and require re‐administration monthly. In contrast, HLA‐matched donor specific VSTs have been detected for over a decade post‐infusion.^83^ Clinical efficacy comparing third party VSTs to donor‐derived, HLA‐matched VSTs show similar clinical efficacy.^81^, ^84^

### 4.3 Manufacturing

VSTs may be single‐valent, designed to target one specific virus, or multivalent and target multiple viruses. There are two broad categories of methods to manufacture VSTs. The first approach is directed selection of existing VSTs from a PBMC collection, and the second is the ex vivo generation and expansion of VSTs.

Directed selection of VSTs relies on isolating T‐cells that are reactive to viral peptides from PBMCs. One strategy involves using synthetic viral peptide‐loaded recombinant HLA multimers to capture T‐cells based on the ability of their TCR to bind specific HLA‐peptide complexes. The drawback of this approach is that it mostly uses HLA class I molecules, thereby enriching CD8+ T cells at the expense of CD4+ T cells.^73^ An alternative approach relies on interferon gamma (INFγ) that is released from VSTs in the presence of viral peptides. Using anti‐IFNγ antibodies, INFγ is affixed to the cell surface of the desired VST, thereby allowing for immunomagnetic column‐based capture. Both CD4+ and CD8+ VST subpopulations are isolated.^85^ Kits such as the CliniMACS Cytokine Capture System (Miltenyi Biotec, Bergisch Gladbach, Germany) allow for GMP‐compliant closed system manufacturing in this way. Others have used antigen stimulation followed by enrichment via cell selection of surface activation markers (e.g., 4‐1BB).^86^ Overall, these direct selection approaches yield few VSTs with limited or no ex vivo expansion prior to infusion. They rely on robust in vivo expansion, prior donor exposure, and are only possible for viruses that form large memory T‐cell pools (e.g., CMV and EBV).^87^

Ex vivo expansion of VSTs generates larger number of cells with decreased alloreactivity by selectively stimulating T cells with viral peptides.^88^ Viral lysates or viral peptides spanning antigenic epitopes of target viruses (e.g., EBNA1 and LMP2 for EBV) are used as a source of antigen along with aAPCs and/or cytokines.

Traditional ex vivo generation and expansion of VST is laborious and requires PMBCs, aAPCs (e.g., dendritic cells, B cells, phytohemagglutinin blasts or genetically modified K562s) and costimulatory cytokines (e.g., IL‐2, IL‐4, IL‐7).^69^, ^89^ This process can take several months and is not readily scalable.^89^ Another approach that uses UCB as a source of T‐cells, aAPCs (UCB‐derived DCs and lymphoblastoid cell lines), pepmixes and cytokines obviates the need for donor seropositivity to the virus(es) in question. However, this process requires months‐long expansion and yields limited numbers of cells for repeat dosing, despite scaling up from 24‐well plates into Grex10 bioreactors (Wilson Wolf, St Paul, MN).^68^

Alternative methods of ex vivo expansion avoid the use of aAPCs and the need for prolonged incubation. PMBCs are incubated with viral pepmixes and pro‐survival cytokines (e.g., IL‐4 and IL‐7). This method allows for polyclonal (CD4+ and CD8+), multivalent VST production in 10–12 days.^74^ Some have developed protocols using open manipulation under GMP conditions to incubate fresh PBMCs with pepmixes, cytokines and culture media before transfer to a bioreactor for a 9–11‐day expansion.^78^ Ultimately, there is increasing desire to transition away from labor‐intensive, manual, open methods to closed GMP‐compliant systems as feasible.

### 4.4 Testing

Many of the lot release criteria for VSTs mirror other cell therapy products: viability, sterility, endotoxin and mycoplasma. Identity assays rely on flow cytometry to enumerate cells with a T‐cell phenotype (e.g., CD3+, CD4+, CD8+), with or without HLA typing. Additional cytotoxicity safety assays with a criterion of a minimum threshold of alloreactivity against recipient/haploidentical cells (e.g., <10% alloreactivity) have been used.^65^, ^78^ Potency tests measuring INFγ released due to viral peptide exposure in enzyme‐linked immunospot (ELIspot) assays are often used and can be helpful in selecting between two equivalently matched HLA VST products in an allogeneic bank.^66^, ^78^ There is variability in release criteria among centers.

### 4.5 Clinical applications

Initial success in preventing and treating CMV, EBV and adenovirus infections in HSCT and SOT patients encouraged exploration of VSTs to treat BKV, HHV‐6, JC virus and SARS‐CoV‐2 infections. There have been high reported rates of response and low adverse events, but a major limitation has been the heterogeneity in study design, patient population and product characteristics.^64^, ^65^, ^80^, ^83^ One study found a response rate of about 2/3 for CMV, EBV and ADV, with a 45% response rate for BKV viremia in the setting of SOT. However, none of the patients with BKV nephropathy had an improvement in their glomerular filtration rate (GFR), suggesting that BKV‐induced damage may be irreversible.^65^ Overall response rates from studies range from 65% to 100% for CMV, 50% to 90% for EBV and 75% and 86% for ADV.^70^

VST therapy is also used in EBV+ PTLD where other approaches fail.^81^ Complete remission or sustained partial remission in HSCT recipients with PLTD to EBV‐VSTs was 68% in one study.^90^ The JC virus may be another target for VSTs. Reactivation of the human polyomavirus JC virus causes a fatal demyelinating disease called progressive multifocal leukoencephalopathy (PML) in the setting of immunosuppression. Treatment options are limited, and a recent review examined 34 patients treated with JC‐VSTs: 21 showed favorable clinical course with an improvement in 16 and disease stabilization in five patients.^91^

Hundreds of patients over dozens of clinical trials (mostly phase I/II with a few multicenter phase III) have been treated with VSTs.^70^ Work is underway to enhance the activity of VSTs using various gene modification strategies, including creating CAR‐VST therapy.^92^ Ongoing advances are expected to enhance the efficacy of VSTs and expand their clinical applicability.

## 5 TUMOR INFILTRATING LYMPHOCYTES

### 5.1 Biology

The goal of TIL therapy is to infuse T‐cells cultured and expanded from a resected tumor, thereby amplifying a patient's anti‐tumor response beyond what can be naturally achieved in vivo. Within a tumor, T cells bearing T‐cell receptors (TCRs) specific for tumor‐associated antigens (e.g., neoantigens, differentiation antigens) become activated and proliferate. Thus, the frequency of tumor‐targeting T‐cells is much higher within a tumor than in peripheral blood.^93^ TILs include polyclonal subsets of CD8+ cytotoxic T‐cells, CD4+ helper T‐cells, and occasionally NK/T‐cells and Tregs. Tumor control is achieved by TILs through direct cytotoxicity and cytokine‐mediated immune activation.

The first early success with TIL therapy dates to the work done by Steven Rosenberg's group at the National Cancer Institute. They provided proof of principle that T‐cells could be expanded ex vivo from a patient's resected tumor (melanoma) and then reinfused into the patient.^94^, ^95^, ^96^ Further work showed that TILs were most effective when administered to a patient who received lymphodepleting chemotherapy and sustained with a course of high‐dose IL‐2.^97^, ^98^ Higher absolute counts of infused cytotoxic CD8+ cells in TIL products are often associated with greater clinical efficacy^99^; however, expansion of the CD4+ TIL subset demonstrates anti‐tumor reactivity in vitro and is hypothesized to play a role in vivo as well.^100^

A challenge with TIL therapy has been that there are often limited numbers of tumor‐targeting lymphocytes with the tumor. The population is heterogeneous and likely contains bystander and suppressive cells (e.g., T‐regs, myeloid‐derived suppressor cells) that inhibit anti‐tumor activity. Other limitations include inefficient engraftment, proliferation and persistence, as well as limited effector functions and dependence on cytokines such as IL‐2.^101^ Tumors with a high mutational burden are choice targets for TIL therapy, but a recent study showed that neoantigen‐reactive T cells tend to show an exhausted cell phenotype.^102^ Indeed, inhibitory effects of the tumor microenvironment remain a considerable barrier.

### 5.2 Starting material

TIL are obtained from surgical harvest of a tumor of sufficient volume and viability, usually a metastasis. TIL expansion from necrotic or heavily pretreated samples is less successful. Healthy, non‐tumor tissue should be excluded as much as possible from further processing. TIL therapy is currently almost exclusively autologous, although there are efforts aimed at creating off‐the‐shelf allogeneic products.^103^

### 5.3 Manufacturing

While the specific protocols used in TIL manufacturing are highly variable, the process generally starts with mechanical disruption and, in many cases, enzymatic digestion of tumor tissue to release lymphocytes. The initial TIL outgrowth phase, known as the pre‐rapid expansion protocol (pre‐REP) involves culture with high‐dose IL‐2. This allows for outgrowth of a polyclonal TIL population over several weeks.

The original pre‐REP (“standard/selected”) TIL protocol involves expansion from tumor cell fragments in multiwell plates forming multiple (typically 48) mini‐cultures, followed by confirmation of activity against autologous tumor cells with immunoassays (e.g., INFγ ELIspot). Only TIL mini‐cultures able to recognize tumor cells in vitro are selected for further expansion.^104^ This process takes 3–5 weeks, generates tens of millions of cells, and increases the 24‐well culture plate number from two primary plates to between 60 and 100 24‐well plates.^105^ This is the most labor‐intensive and contamination‐prone part of the protocol, but aspects such as repeated media changes lend themselves to automation.^106^ The alternative, young TIL pre‐REP protocol does not select specific tumor‐reactive lymphocytes but rather expands all outgrown TILs. The advantages of this method include a shortened expansion phase (10–14 days) and the generation of a less differentiated product exhibiting greater proliferative capacity and a more polyfunctional phenotype.^107^

Cells from either pre‐REP protocol are subsequently expanded into clinically relevant doses of billions of cells using a REP protocol. Reactive TILs from the selected pre‐REP protocol are pooled. TILs are expanded in the presence of IL‐2, with some protocols incorporating supplementary cytokines, other agonists such as anti‐CD3 (OKT‐3) antibodies, and irradiated feeder cells. Examples of irradiated feeder cells include autologous/allogeneic unmodified PBMCs, autologous myeloid‐derived dendritic cells, and autologous lymphoblastoid cell lines (LCLs).^105^, ^106^ The REP phase typically occurs for 2 weeks and begins in multiple T175 flasks before transferring into gas‐permeable culture bags.^105^ Containers such as the Grex have greatly aided in scale‐up efforts to generate sufficient cells for clinical use.^105^, ^106^ Academic TIL products are often infused fresh, while commercially manufactured products are cryopreserved and later thawed for infusion.

Many modifications to the material inputs and culture conditions have been made to the above schema. For example, additional cytokines beyond IL‐2 and agonistic antibodies (e.g., anti‐4‐1BB) can be used.^103^, ^105^ By using whole exome and RNA sequencing of tumor cells, it is possible to identify candidate neoantigens that can then be synthesized as peptides for loading onto aAPCs used in TIL cultures.^106^ This offers another way to selectively expand tumor‐reactive lymphocytes. Approaches such as these introduce additional complexity and cost, can further lengthen manufacturing turnaround time, and present challenges in efforts to scale manufacturing.

### 5.4 Testing

Standard lot release criteria include viability, sterility (culture +/− gram stain), endotoxin and mycoplasma. Identity testing involves flow cytometry assays for T‐cell markers. Some authors advocate confirming the presence of tumor‐reactive clonotypes in the TIL product by sequencing the TCR repertoire of the drug product (DP).^108^ As tumor cells were part of the starting material, it is important to exclude the presence of tumor cells in the DP by cytopathology or other methods. Potency testing often involves tumor‐specific T‐cell activation (e.g., INFγ ELISA). For an in‐depth review of lot release criteria for TIL products, we point readers to the excellent review by Lievense et al.^108^

### 5.5 Clinical applications

Momentum in the TIL field was slightly overshadowed by the advent of immune checkpoint inhibitors (ICI).^103^, ^109^ Nevertheless, after decades of research, the FDA granted accelerated approved to the first TIL therapy (lifileucel, Iovance) for the treatment of unresectable/metastatic melanoma previously treated with a PD‐1 blocking antibody. At 5 years, the overall response rate (ORR) in 73 patients who received lifileucel was 31.5% with a median duration of response of 36.5 months. Median overall survival (OS) was 13.9 months with 5‐year OS of 19.7%.^110^

Beyond melanoma, encouraging results have been found in non‐small cell lung cancer (NSCLC), cervical cancer, breast cancer and gastrointestinal cancer. Early trials in the setting of NSCLC show 21%–26% response rates in ICI‐pretreated NSCLC. Some durable complete responses occurred in EGFR‐mutated, never‐smoker patients, a cohort that is often resistant to ICIs.^111^, ^112^ The C‐145‐04 phase II multicenter trial demonstrated an ORR of 44% in patients with ICI‐naive advanced cervical cancer that progressed after at least 1 prior line of chemotherapy.^113^ Based on these findings, FDA granted breakthrough designation status to this product.

Next generation TIL strategies aimed at enhancing specificity and function are underway. For example, neoantigen‐specific CD8+ cells can be captured from peripheral blood of patients using neoantigen‐HLA multimers created based on data generated from whole exome and RNA sequencing.^114^ Another strategy at enhancing specificity involves selecting CD8+ TILs co‐expressing certain surface proteins (e.g., CD39 and CD103).^115^ Approaches aimed at enhancing TIL function include engineering costimulatory receptors, altering inhibitory receptors/immune checkpoints (e.g., PD‐1 and CISH proteins), expanding stem‐like T‐cells, and engineering IL‐2 independence.^103^, ^106^, ^116^

## 6 CONCLUSION

CT has only grown as a significant therapeutic modality in medicine with the FDA approval of the first CAR‐T in 2017. Since then, other types of IECs, such as other types of T cells (i.e., TILs and TCR) and MSCs, have also received FDA approval, with many more cell types expected to be licensed and more broadly accessible to patients in the coming years. While the starting materials, manufacturing steps and the testing of the various cell types is variable, these cells share the general approach to clinical translation which begins with evaluating the pre‐clinical studies, and is followed by scale‐up, optimization, and methods validation before the products reach patients in early phase trials. CT labs, many of them housed within TM programs, will continue to play a major role in advancing the clinical translation and manufacturing of IECs.

## CONFLICT OF INTEREST STATEMENT

The authors have disclosed no conflicts of interest.

## Data Availability

Data sharing not applicable to this article as no datasets were generated or analysed during the current study.
